# Sustained resilience as a unifying property of cities and the built environment

**DOI:** 10.1038/s42949-026-00457-3

**Published:** 2026-08-25

**Authors:** Stergios-Aristoteles Mitoulis, Nadiia Kopiika, Roberta Di Bari, Sotirios Argyroudis

**Affiliations:** 1 Centre for Global Infrastructure Resilience, The Bartlett School of Sustainable Construction, UCL, London, UK; 2 MetaInfrastructure.org, London, United Kingdom; 3 https://www.BridgeUkraine.org; 4 Brunel University of London, Uxbridge, UK

**Keywords:** Ecology, Ecology, Environmental sciences, Environmental social sciences, Environmental studies

## Abstract

The built environment evolves, deteriorates and regenerates over time, yet resilience is commonly reduced to discrete event-based recovery while long-term performance is treated through sustainability. This separation obscures how resilience unfolds across lifecycles. We introduce sustained resilience as a unifying property that integrates resilience and sustainability, capturing the cumulative and adaptive capacity of the built environment and cities to maintain function, learn from disruption and evolve through successive cycles of change.

## Introduction

Like biological systems, the built environment consumes resources, produces waste, interacts and is influenced continuously by its surroundings. While the built environment is not a living organism in a biological sense, this analogy highlights its dynamic, adaptive and evolving nature^1^. It is therefore not a static backdrop to human activity but a dynamic system of interdependent structures and processes as it continuously interacts with society and nature. Conceptually, this perspective is grounded in systems thinking and the understanding of cities as complex Social-Ecological-Technological Systems (SES/SETS). Within social-ecological systems research, Panarchy theory conceptualises complex systems as nested adaptive cycles of growth, conservation, release and reorganisation, operating across spatial and temporal scales^2,3^. Cities exhibit many of these characteristics, evolving through cycles of growth, decline, adaptation and renewal while responding to changing environmental, social, economic and technological conditions. The accelerating pace of climate change and the intensification of natural and anthropogenic hazards (Supplementary Information 1) have exposed persistent vulnerabilities in buildings, infrastructure and cities, underscoring the need for resilience across the lifecycle^4,5^. While construction and urban development contribute substantially to global greenhouse gas emissions, they are also disproportionately impacted by disruptive events, resulting in escalating recovery costs and long-term serviceability challenges^6^. This dual role has elevated resilience and sustainability as essential guiding principles for the future of the built environment.

Traditionally, resilience has been defined as the capacity of systems to anticipate, absorb and recover from disruptions while maintaining functionality^7,8^. Sustainability, rooted in the Brundtland Report^9^, emphasises meeting present needs without compromising the capacity of future generations, and has been operationalised through frameworks such as the UN Sustainable Development Goals^10^. While these definitions remain foundational, both sustainability and resilience scholarship have evolved considerably over recent decades. Contemporary sustainability research increasingly conceptualises sustainability as a dynamic property of interconnected social, ecological, technological and governance systems operating within environmental limits and societal objectives, while resilience has expanded beyond recovery from disturbances to encompass adaptation, transformation, and cross-scale system dynamics. Recent perspectives emphasise planetary boundaries, social equity, adaptive capacity and transformative change as essential dimensions of sustainable development^11–13^. This evolution has shifted both concepts away from predominantly resource-efficiency and event-recovery paradigms towards systems-oriented perspectives concerned with the long-term capacity of coupled human and natural systems to persist, adapt and thrive. As a result, the two concepts have often been treated separately: resilience as short-term and event-focused, e.g. REDi^14^, sustainability as long-term and resource-oriented^15^. However, urban systems scholarship has also shown that this separation is incomplete, because sustainability, resilience and transformation are interlinked across spatial and temporal scales depending on what is sustained, for whom and over what timeframe^13^. Early contributions aimed to bridge this divide, by advancing a shared language for lifecycle performance^16^. Society-based perspectives highlighted well-being as the ultimate goal of resilient and sustainable infrastructure^17^, while an ongoing debate questions whether sustainability and resilience should remain distinct or be combined^18^, suggesting both complementarity and tension. More recent work has aimed to resolve this tension. A conceptual synthesis of resilience and sustainability in the built environment^19^ provides a framing of the two properties as distinct yet interdependent paradigms. An alternative integration was proposed later, that linked resilience and sustainability metrics across natural hazards and climate risks^20^ in built environments^21^. Built-environment resilience research has also moved beyond single-hazard and event-based perspectives toward more integrated approaches addressing multiple crises, adaptability, equity, inclusivity, governance and institutional capacity^22,23^. These advances provide a foundation for understanding resilience as a multidimensional and cross-sectoral property of the built environment.

Although recent literature has made substantial progress toward integrating resilience and sustainability, many studies continue to treat them as parallel agendas or assess them using different temporal and methodological perspectives. Resilience is commonly reduced to event-based recovery, while the long-term performance and evolution of the built environment are primarily captured through sustainability, fragmenting how system resilience is understood over time. This disjointed and temporally incompatible treatment creates a critical gap by lacking an integrated framework for quantifying resilience across the full lifecycle. As a result, policy blind spots exist due to the absence of a single method capable of capturing how living built environments sustain performance across their lifecycles. A new method that complements existing frameworks must redefine performance measurement to capture how buildings, cities and services evolve, adapt to shifting demands and policy regimes, and remain functional. The challenge lies in designing methods that can operationalise this spatiotemporal integration, quantifying resilience and sustainability as dynamic and mutually reinforcing dimensions over time, across services and across scales. Addressing this challenge requires frameworks that integrate threat-agnostic resilience^24^—focusing on adaptation and recovery under both known and unknown disruptions—with lifecycle sustainability assessment, capturing evolving environmental, social and economic outcomes across time and scale, inclusivity aligned to SDGs and societal expectations^25^.

## Sustained resilience across scales

The escalating frequency of climate-linked disasters and the systemic vulnerability of the built environment have created an urgent imperative to move beyond conventional practice. Regulatory requirements for cities and infrastructure now demand not just resilience to shocks, but sustained performance over extended lifecycles^21^. However, a persistent dichotomy remains. While the built environment functions as a dynamic system of interdependent structures, current assessment frameworks historically treat resilience - often narrowly defined as shock absorption and recovery^26^ - as distinct from sustainability’s focus on long-term resource efficiency and equity^27^, although recent scholarship has increasingly sought to integrate these concepts across social, ecological and technological domains^13,21,28^. This distinction remains evident in leading assessment systems such as DGNB^29^ and BREEAM^30^ and can lead to conflicting priorities in practice^31^, where advancing one goal often inadvertently creates barriers to the other^32^. This approach fails to capture the dynamic nature of the built environment in the face of intensifying human-induced^33^ and natural hazards^4,5^.

The integration of concepts at the city scale has evolved toward a recognition of their inherent synergy^34,35^. Historically, frameworks for city assessment treated resilience as shock absorption, reinforcing a conceptual separation from sustainability agendas^36^. This lack of integration has often led to conflicting outcomes in practice, where efforts to advance one goal inadvertently created barriers to the other. Recent global crises have further exposed the risks of this disconnect, underscoring the need for approaches that address both acute shocks and systemic vulnerabilities^37^. For example, foundational frameworks often prioritise social and infrastructural resilience dimensions while offering limited consideration of environmental or economic sustainability^38,39^. This sector-specific focus is evident in studies targeting singular hazards^40,41^, or climate extremes^42^, which overlook the potential for systemic cascading failures^43^.

Recent urban resilience research has increasingly adopted Social-Ecological-Technological Systems (SETS) perspectives, recognising cities as interconnected social, ecological, technological, institutional and economic systems. However, significant challenges remain in operationalising and quantifying the interactions among these domains across spatial and temporal scales^44^. For instance, while foundational works by the Resilience Alliance^45^ and Meerow et al.^46^ provide crucial conceptual grounding, they offer limited explicit linkage to the physical systems underpinning city functionality. Similarly, frameworks synthesised for climate-responsive cities often lack integration of sustainability principles across the entire lifecycle^47^. Methodological reviews conclude that resilience research seldom operationalises sustainability parameters^48^, a disconnect confirmed by empirical comparisons of cities where institutional resilience is often prioritised over infrastructural sustainability^49^. Furthermore, prevailing urban research relies predominantly on static, cross-sectional models. Comparative indices^50^ and capacity-based frameworks^51^ offer benchmark assessments but fail to account for dynamic, adaptive processes. This limitation is reflected in contemporary monitoring tools such as the EU Regional Resilience Dashboard, which provide valuable regional benchmarking but remain temporally discrete and indicator-aggregated rather than lifecycle-dynamic^52^. These approaches provide diagnostic profiles at discrete moments but inadequately represent how cities learn, reorganise and transform over time in response to chronic stresses - a capacity central to the ‘living organism’ metaphor^53^.

Responding to these challenges, the evolving state of the art is defined by deliberate efforts to develop integrated frameworks. Scholars have advocated for systems-thinking perspectives that bridge domains across sectors, scales and temporal horizons^54,55^. Sustained resilience adopts a SETS perspective, recognising cities as complex adaptive systems whose resilience emerges from interactions among social, ecological, technological, institutional and economic domains^56,57^. This perspective is compatible with adaptive-cycle and Panarchy thinking^2^, which emphasise cross-scale system dynamics and self-organisation^58^. Accordingly, the framework extends beyond grey infrastructure performance to encompass social cohesion, equity, public health, ecosystem services, cultural values and adaptive governance, while providing a lifecycle-based structure for evaluating their combined performance over time^44^. However, sustained resilience differs in purpose as it operationalises these dynamics as a lifecycle-based property for assessing, through a quantitative mathematical framing, whether built environments maintain, lose, or improve desirable infrastructural, services, societal and connectivity performance over time.

Policy-oriented guidance, such as the World Bank’s Handbook for Livable and Resilient Cities^59^, reinforces the need for structured, lifecycle-aware resilience governance. This shift is evident in applied frameworks examining nature-based solutions as co-beneficial strategies^60^ and data-driven models like Zhang et al.’s MetaCity concept^61^, which leverage analytics to dynamically allocate resources in pursuit of sustainability targets. However, while ‘smart city’ paradigms offer holistic models aligning technology with governance^35^, they risk exacerbating socio-economic inequalities^62^ and require multi-criteria evaluation to ensure regulatory feasibility^63^. Ultimately, a persistent implementation gap remains: while academic models endorse transformation, municipal policy frequently defaults to narrow, hazard-specific interventions due to institutional path dependencies^64^.

Due to the demonstrable exacerbation of climate-linked natural disasters and the systemic vulnerability of existing assets, there is a growing imperative to move beyond conventional practice toward holistic lifecycle integration^65^. At the individual asset level, attempts to integrate sustainability and resilience have been made for buildings^66^, bridges^33^ and critical infrastructure^21^. Yet, significant methodological disagreements persist. Resilience of the built environment is typically assessed through the ‘4 R’ framework - robustness, resourcefulness, redundancy and rapidity^67^ - or through flexibility and adaptability characteristics^24^. Conversely, environmental sustainability is primarily assessed through Life Cycle Assessment (LCA), focusing on Global Warming Potential (GWP) and other impact categories^68^. Economic sustainability aligns with Life Cycle Costing (LCC), while resilience-focused economic analysis shifts to direct and indirect costs associated with failure^69^. Social sustainability remains the least developed dimension, often limited to minimising negative impacts such as downtime^70^.

These two crucial pillars of long-term asset performance continue to operate in many cases as separate disciplinary silos. Despite substantial progress toward integration, resilience and sustainability assessments are often developed using different metrics, temporal scales and disciplinary traditions. This confusion translates into two major barriers: the timeframe of assessment and the consideration of interdependencies. Regarding timeframe, resilience studies often prioritise ‘bouncing back’ quickly to minimise short-term impacts^71^. In contrast, sustainability metrics are quantified over the asset’s lifecycle, implicitly assuming code compliance equates to resilience^72^. Regarding interdependencies, existing approaches often focus on narrow interactions, such as how repair times influence economic costs^73^ or how structural robustness impacts GWP^74^. Comprehensive quantification of synergistic and trade-off relationships remains rare, leading to risks of double counting benefits^75^.

A final and critical methodological barrier is the accurate modelling of asset performance evolution. Resilience engineering has advanced lifetime resilience concepts that account for progressive deterioration and hazard events^76^. However, standard LCA approaches rarely account for dynamic decline, operating on simplifying assumptions of static performance^77,78^. This failure to model deterioration means that environmental impacts, such as operational energy or maintenance frequency, are often underestimated over the asset’s functional duration. Emerging paradigms are beginning to address these gaps. A conceptual synthesis^19^ frames the two properties as distinct yet interdependent. Integrated frameworks for dynamic built systems are being proposed to model adaptive capacity^4^, while the Climate Resilience Index^20^ advances assessments by linking hazard, exposure and sustainability outcomes. Table 1 reveals growing efforts toward integration, yet comparatively few frameworks bridge the concepts methodologically for jointly evaluating resilience and sustainability across multiple spatial and temporal scales in the built environment.

**Table 1:** Sustained resilience: bridging the gaps in built environment resilience and sustainability frameworks

Framework/Study	Core concept and innovation	Innovation and complementarity of Sustained Resilience	Examples of operationalisation and application
Gunderson and Holling^2^; West et al.^3^	Panarchy and adaptive cycles explain how nested systems evolve through growth, conservation, release and reorganisation, with cross-scale ‘revolt’ and ‘remember’ dynamics	Sustained resilience builds on Panarchy by translating cross-scale adaptive dynamics into a lifecycle-based decision and quantification framework for built environments and cities	Assess whether repeated disruption–recovery–renewal cycles lead to cumulative improvement, stagnation, lock-in or decline in urban services and infrastructure performance
Elmqvist et al.^13^	Links urban sustainability, resilience and transformation through cross-scale developmental trajectories; distinguishes normative sustainability vs non-normative resilience and transformation as pathways change	Contributes to ‘resolving tensions’ with a conceptual integration by translating this trajectory into a lifecycle property → Sustainability and resilience are unified into sustained resilience (single model), which is quantified across time, space, services and tiers	Compare for flood adaptation options by jointly tracking service continuity, emissions, ecosystem services over e.g. short- (1 year), medium- (3 years), long-term (50 years)
Hewidy et al.^34^	Distinguishes urban resilience (crisis recovery) from sustainability (resource protection) in planning (focus on Finland)	From static, cross-sectional assessments toward a dynamic, unified discipline by bridging different domains → Integrate physical + social domains across lifecycles and renewals	Evaluate post-disaster urban regeneration strategies balancing rapid recovery, resource efficiency, community wellbeing and infrastructure performance over lifecycle
Zhang and Li^31^	Separates urban resilience and urban sustainability, flags overlap as weakness and potential conflict	Discusses latent parts of sustained resilience → Treats overlaps as the foundation for integration (see Fig. 1)	Calculate across spatiotemporal scales to assess whether transport investments jointly enhance resilience and sustainability
Castaño-Rosa et al.^22^; Tähtinen and Toivonen^23^	Multi-crisis built-environment resilience through adaptable, equitable and green infrastructures, enabled by governance and institutional capacity	Operationalises dimensions as performance trajectories and cumulative assessments across temporal and spatial scales	Track and measure equitable access, governance capacity, green infrastructure and service continuity through repeated climate, health and energy crises
Buzási and Csizovszky^32^	Reviews sustainability, resilience and lock‑in effects; finds lack of combined rigorous analyses	Complements existing framework to fill the capability gap with sustained resilience by coupling long‑term spatiotemporal and service interactions to mitigate lock‑ins	Choose optimal district redevelopment pathways to rectify lock-ins by assessing cumulative social, environmental and economic over time
Zhang et al.^61^	Data‑driven resource/decision MetaCity framework for city optimisation (simulation + multi‑modal data)	Demonstrates bottom-up and top-down data use and optimisation → Treatment of evolving sustained resilience through repeated decline–failure–recovery–prosperity cycles, captured by new performance archetypes (Fig. 2)	Optimise city investments using evolving for sectors underpinning mobility, energy, accessibility and resource allocation to minimise impacts
Di Bari et al.^21^	Two‑step integrative indicator-based method for asset‑level evolutionary buildings’ performance vs hazards	Proposes dynamic asset modelling → Upscaling to interconnected system lifecycles, e.g. cities (see Fig. 3, Box 1)	Assess whether adaptive building retrofits improve lifecycle functionality, emissions, costs and resilience under multiple hazards
Kopiika et al.^33^	Scoring framework to prioritise bridge recovery in conflict zones considering resilience and environmental impacts	Dynamic prioritisation for post‑event repair → Tracking of temporal evolution before, during, after events, upscaled from buildings and assets to districts and cities (Box 1)	Prioritise post-disaster city reconstruction projects using short-, medium-, long-term impacts on accessibility, equity, recovery speed and environmental performance
Sánchez-Silva et al.^19^	Conceptual review on resilience + sustainability trade-offs and decision challenges	Facilitates diagnosis of needs and paths → Operationalises a unified method for assessment of performance across lifecycle	Quantify trade-offs between rapid recovery, lifecycle costs, carbon emissions and long-term housing provision for urban residential areas
DGNB v. 2023^29^	LCA/LCC sustainability; resilience as economic/functional durability via checklist scoring	Aggregates lifecycle metrics and treats the built environment as dynamic and evolving system → Treats evolving functionality across cycles accounting for disruptions and perturbations	Evaluate if resilient design measures improve lifecycle performance without increasing emissions, costs, or resource consumption across temporal scales
BREEAM v.7^30^	Distinguish resilience (4R) and sustainability (LCA/LCC); service life central to impact	Dynamic lifecycle aggregation of both properties → Embedding temporal continuity and system interactions	Compare and apply climate adaptation measures by tracking functionality, emissions, maintenance and service continuity throughout asset lifecycles
Fang^90^	Doughnut-shaped safe and just space framework: trade‑offs between social goals and planetary boundaries	Provides method for quantification of trade-offs → Normalises and links social + environmental aims across lifecycle	Quantify trade-offs (e.g. Pareto) between reducing displacement and maintaining emissions within city/country/continent/planetary-boundary constraints during climate adaptation planning and implementation
Lai and Zhao^91^	Smart‑city research involving technology-driven and human-centred approaches	Quantifies divergence risk in resilience and sustainability approaches → Bridges multiple domains beyond technical and human in a multi-tier framework across spatial and temporal scales	Assess and select smart-city interventions using combined metrics of accessibility, equity, energy use, governance and resilience trajectories
Cai et al.^92^	Descriptive link between US smart city programmes and local SDG indicators; resilience is included within SDGs	Observational SDG links → Supports a dynamic mechanism showing how iterative resilience cycles underpin SDG outcomes and quantitative dynamic sustained resilience across city lifecycle	Quantify the long-term contribution of investments to SDG-aligned outcomes across social, environmental and economic dimensions. Capturing the upscaling/downscaling of : assets ↔ cities/regions ↔ countries ↔ planet

## From resilience and sustainability to sustained resilience

**Fig. 1:**
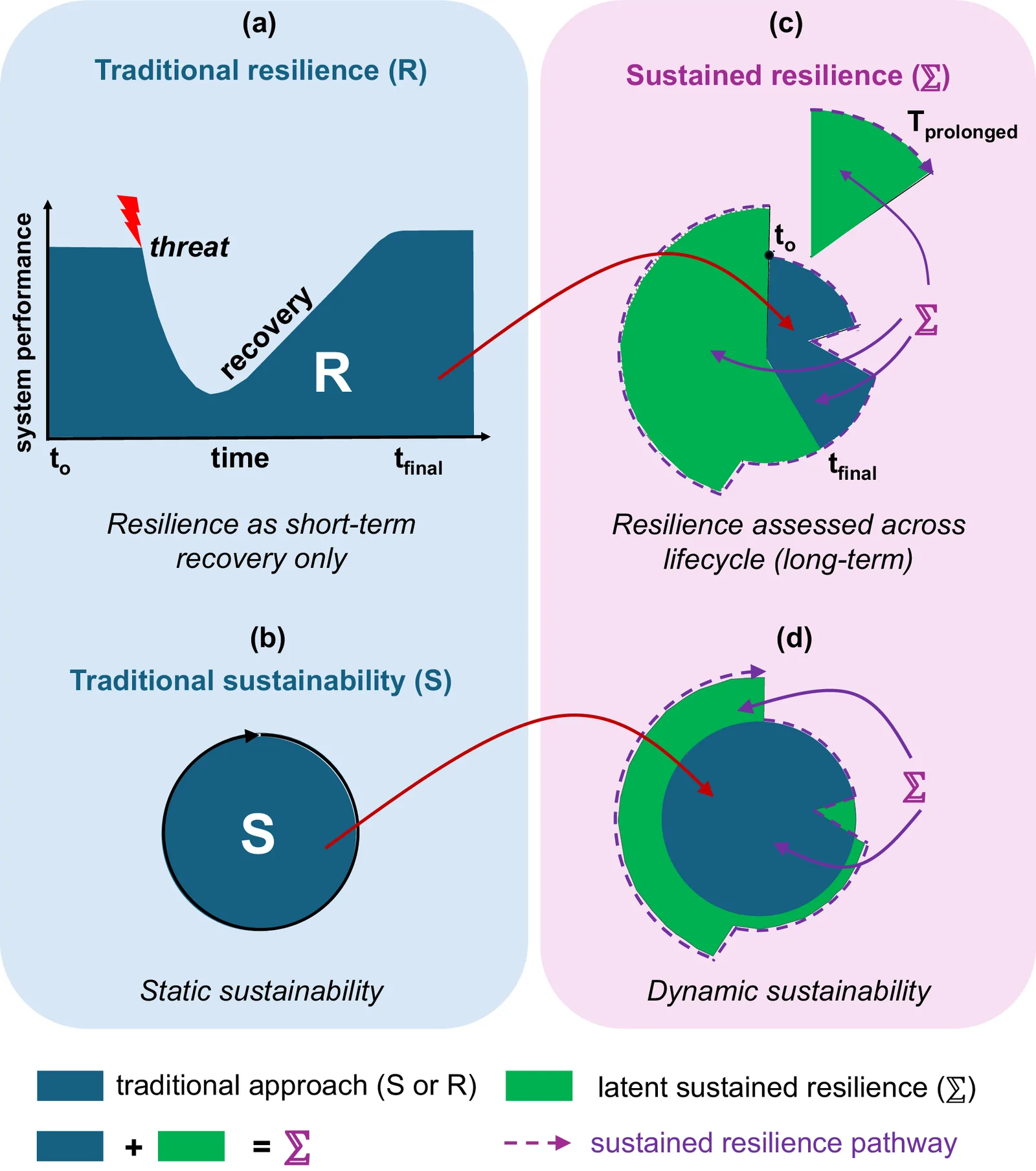
From traditional resilience and sustainability to a single property: sustained resilience . The conceptual shift from conventional assessments of resilience and sustainability to the unified concept of  across the lifecycle of the built environment. **a** Traditional resilience (R), commonly assessed within a short, event-bounded time window, where performance drops following a threat and recovery is evaluated only up to a predefined horizon. This framing captures short-term recovery but typically excludes long-term deterioration, adaptation and repeated disruptions. **b** Traditional sustainability (S), commonly treated as a static lifecycle property assessed through aggregated indicators (e.g. emissions, cost and resource use), but generally disconnected from disruption and recovery processes. **c** Sustained resilience  derived from resilience, where performance is assessed across the full lifecycle or urban epoch and may be prolonged through adaptation, upgrading, recovery and renewal. **d** Sustained resilience  derived from sustainability, where sustainability is treated as a dynamic, disturbance-sensitive process that evolves through successive cycles of use, disruption, recovery and adaptation. The dark blue areas represent performance typically captured by conventional resilience or sustainability assessments, while the green areas represent latent sustained resilience—the additional accumulated, time-integrated performance realised across the lifecycle. Red arrows indicate the conceptual transition from conventional resilience and sustainability assessments to sustained resilience. Purple arrows and dashed purple pathways indicate the evolution of performance through time, including adaptation, renewal, and lifecycle extension. t_0_ denotes the beginning of the assessment period, t_final_ denotes the original lifecycle or urban epoch over which performance would conventionally be evaluated and T_prolonged_ denotes the extended lifecycle or epoch achieved through adaptation, renewal, upgrading, or recovery interventions. Integration of resilience and sustainability therefore yields a unified lifecycle-oriented property of the built environment——representing total realised performance over time.

The evolving literature presented above demonstrates a clear shift from siloed approaches to integrative methodologies that view resilience and sustainability as mutually reinforcing dimensions. While numerous resilience and sustainability frameworks have successfully integrated governance, adaptation, social-ecological dynamics and infrastructure performance, relatively few provide a common lifecycle-based structure through which resilience and sustainability outcomes can be jointly evaluated over extended temporal horizons^22,23^. A well-established context, Panarchy, provides the theoretical language of cross-scale adaptive dynamics and adaptive cycles, but it does not explicitly provide a lifecycle-based framework for quantifying the co-evolution of resilience and sustainability. Unlike short-term resilience or stand-alone sustainability, we introduce the concept of *sustained resilience*: a lifecycle-oriented method that unifies resilience and sustainability by quantifying performance across successive cycles of disruption, recovery and renewal to achieve lifecycle continuity, adaptation and measurable co-benefits (Fig. 1). Sustained resilience should be understood as complementary to Panarchy-informed SETS perspectives. It provides a decision-oriented structure for quantifying cumulative outcomes across disruption, recovery, adaptation and renewal cycles, while assessing whether the functions being sustained remain socially, environmentally and economically legitimate over time in increasingly connected cities^79^.

**Fig. 2:**
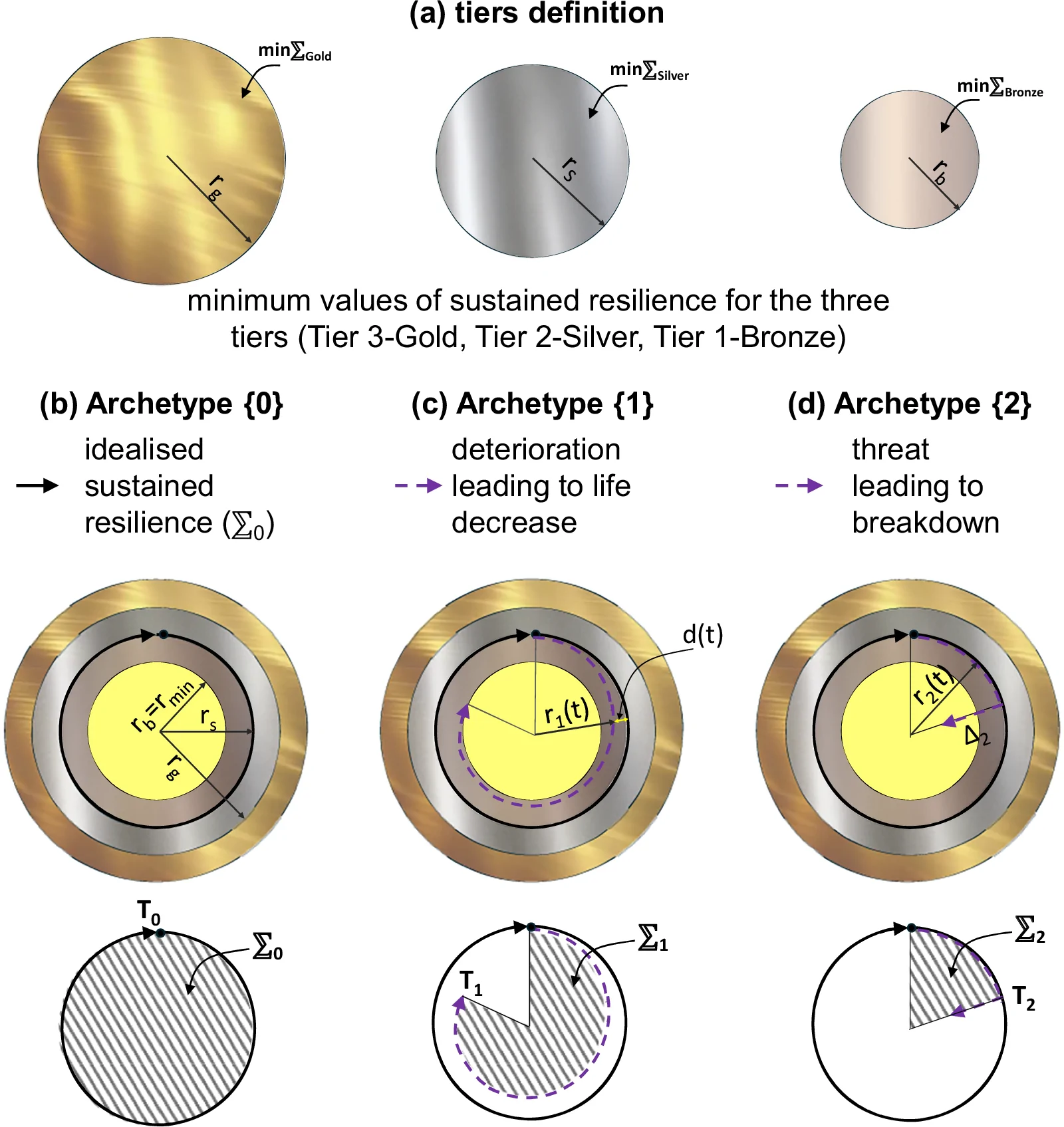

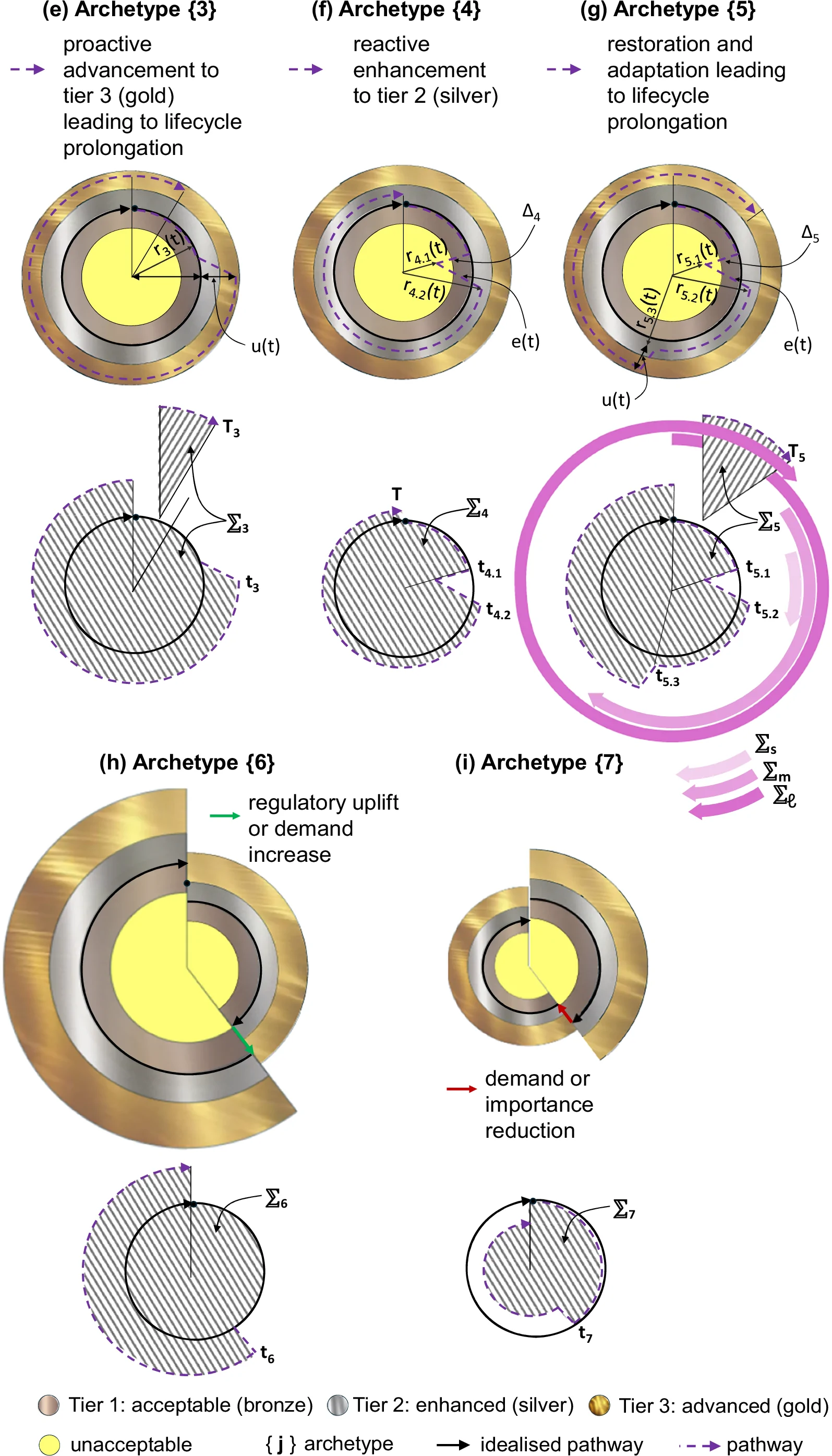
Archetypes of sustained resilience across the lifecycle of built environments. The shaded areas of each one of the eight archetypes represent the realised measure of sustained resilience pathway over the lifecycle, highlighting either reductions from the baseline (idealised pathway) due to deterioration, threats and obsolescence, or gains achieved through proactive, reactive and adaptive interventions. **a** Definition of minimum sustained resilience thresholds for the three tiers: Tier 3 (Gold), Tier 2 (Silver) and Tier 1 (Bronze). **b** Archetype {0}: Idealised baseline trajectory (solid black line) depicts sustained resilience  maintained perfectly over time without deterioration or hazard impacts. **c** Archetype {1}: Deterioration progressively contracts resilience capacity, reducing the lifecycle below its design potential. **d** Archetype {2}: Severe threats or negative changes (internal, climatic, natural, or anthropogenic) causing sudden breakdown and premature loss of the built environment. **e** Archetype {3}: Proactive advancement elevates resilience to Tier 3 (Gold) standard, prolonging lifecycle beyond original design. **f** Archetype {4}: Reactive enhancement follows a disruption, restoring resilience to a specific tier (this could be Tier 1, 2 or 3). **g** Archetype {5}: Restoration and adaptation across successive cycles of hazards or degradation progressively enhancing resilience and extending lifecycle. This is an example of an archetype that captures dynamic sustained resilience across three temporal horizons: short-term,  (months to years); medium-term,  (years to a decade); and long-term,  (the full lifecycle). Resilience at each temporal horizon should meet the agreed tier requirements. **h** Archetype {6}: Regulatory uplift or increased user demands raise performance thresholds, rendering assets obsolete or non-compliant unless renovated or strengthened. **i** Archetype {7}: Demand or importance reduction causes contraction of tier thresholds. The coloured circular diagrams illustrate the conceptual evolution of sustained resilience across the Bronze, Silver and Gold tiers, while the corresponding black-and-white diagrams represent the realised sustained resilience  for each archetype. The yellow region denotes unacceptable performance. Bronze, Silver and Gold regions denote increasing levels of sustained resilience. Solid black arrows indicate idealised traditional pathways, while dashed purple arrows indicate realised sustained resilience pathways. Symbols  denote the sustained resilience associated with Archetypes {0}–{7}; T_0_–T_7_ denote lifecycle or epoch durations; r(t) denotes time-dependent performance; d(t) denotes deterioration; Δ denotes sudden performance loss due to a threat or hazard; e(t) denotes enhancement through recovery or adaptation; and u(t) denotes proactive uplift resulting from planned interventions.

Our framing encompasses eight representative archetypal pathways (Fig. 2), ranging from deterioration and breakdown to proactive advancement and policy-driven obsolescence in multihazard environments. These archetypes operationalise sustained resilience as a measurable systemic property and are mathematically expressed through the formulations presented in the following section. By applying this lifecycle-oriented perspective, we offer a scalable, multi-tiered method applicable across assets, networks and cities, enabling a unifying language for engineers, planners, policymakers and communities by seamlessly integrating resilience and sustainability into one property, *sustained resilience*, as the total realised performance over the lifecycle. Sustained resilience, therefore, requires maintaining present functionality without eroding future adaptive capacity, preventing short-term recovery decisions from generating long-term performance losses. Thus, it requires systems to maintain function under stress and recover rapidly, while simultaneously preserving long-term environmental, social and economic viability.

Table 2 synthesises how sustainability and resilience are defined across global frameworks and practice, and introduces sustained resilience as a lifecycle-integrated concept that links short-term recovery with long-term adaptive performance and sustainability trajectories in the built environment. Several conceptual elements in the table are already established in SES/SETS resilience scholarship; the contribution of sustained resilience is their lifecycle quantification for built-environment performance across assets, services, infrastructure systems and cities.

**Table 2:** Sustainability and resilience vs the conceptual distinctions and innovative framing of sustained resilience

Policy, dimensions & principles	Sustainability	Resilience	Sustained resilience—conceptual distinction and innovation in framing for built environments
Foundational UN definition	Meeting present needs without compromising future generations^93^	Ability to resist, absorb, adapt, recover and transform while maintaining essential functions^94,95^	From single-event recovery to threat-agnosticity^24^ → lifecycle continuity, quantifies cumulative performance over repeated failure–prosperity cycles and new performance archetypes
ISO standard definition	Integrate environmental, social and economic performance over time^96^	Organisational capacity to anticipate, respond, adapt and prosper under change^97,98^	Embed time-integrated resilience thinking throughout lifecycle → short-, medium-, long-term adaptation pathways
Spatial dimension across scales	Lifecycle modules (A1–C4), often at asset → project scale, relying on spatial distribution^21,68^	Assets that anticipate, withstand, recover and adapt^99,100^. Shaped by spatial interdependencies and network effects across regions/cities^18,43^	Integrate spatially distributed lifecycles across SETS, capturing interactions among infrastructure, ecosystems and communities with urban resilience; cross-scale effects: *Bottom up* from component → city and *Top-down*: global → domain (see further in Table of Box 1), convergence to meet sustained resilience goals
Temporal dimension	Resource-efficient, socially beneficial and economically viable across lifecycle^99–101^. Intergenerational (decades to centuries)^21,77^	Immediate, short- and medium-term (days to years), transformational potential with long-term considerations for environmental impacts^71,76^	Seamless temporal integration: assess how adaptations compound into multi-temporal gains or losses, e.g. environment, across lifecycle of assets and city epochs (see Fig. 2, Fig. 3) → lifecycle prolongation + city prosperity
Service & operational dimensions across scales	Long-term equilibrium of environmental, social and economic systems services within planetary boundaries^9,11–13^	Capacity to maintain functionality (i.e. services, operations, capacity) under shocks/stresses^8^	Nested temporal layers: shock absorption → adaptation → short, medium, long-term performance. Unified method across social, ecological, technological, economic and governance domains (e.g. functionality, ecosystem services and continuity)
Environmental dimension	Emissions reduction, resource efficiency and circularity^47,68,74^	Minimise environmental damage from hazards and recovery^47,68,74^	Track environmental resilience over time: cumulative GWP, resource depletion and ecosystem services under repair/adaptation cycles and fluctuating demands
Economic + Governance dimension	Lifecycle cost optimisation, long-term value and intergenerational affordability^69,73^. Stable institutions, long-term planning and policy alignment with sustainability goals^35,37^	Reduce direct/indirect losses from disruptions and downtime and cascading events^33,67^. Preparedness, crisis management and capacity for recovery^35,37^	Cost-at-failure consequence → value-over-lifecycle dynamic framing: avoided losses, payback of investments for , value of decarbonisation, optimal interventions + Adaptive lifecycle governance
Societal dimension	Equity, wellbeing, accessibility and inclusivity^8,38,45,80^	Protect lives, services and community functionality during and after threats^8,38,45,70^	Measure societal prosperity and justice: continuity of services and infrastructure access, equitable distribution of risks and benefits, inclusivity and learning from disruptions under changing socio-political conditions.

## Sustained resilience as a cross-scale property of the built environment

### Archetypes of sustained resilience

Our approach quantifies sustained resilience  as a unified property that integrates sustainability performance across space and time—spanning buildings, districts and cities, from short-term responses to full lifecycles and urban epochs. It represents not just structural or functional performance, but also environmental impacts, economic efficiency, such as cost-effectiveness over time and social inclusion or accessibility, e.g. how many people benefit from the infrastructure. Sustained resilience adopts a people-centric perspective, as it recognises people and communities as both contributors to sustained resilience and also recipients of its benefits. Accordingly, the framework and archetypes consider not only service continuity and infrastructure performance, but can also describe who benefits, who bears risk, whose needs are prioritised and how social cohesion, inclusion, accessibility, institutional trust and adaptive capacity evolve over time. Thus, our framing can be used not only for infrastructure but also for archetypal social dimensions of sustained resilience. For example, a city may rapidly restore transport, energy and water services after a flood and therefore appear resilient according to conventional infrastructure-based metrics. However, from a sustained resilience perspective, recovery should also be evaluated through social indicators such as displacement, livelihood restoration and accessibility^80^. If these dimensions continue to deteriorate despite successful infrastructure recovery, the social domain may follow a declining archetype, reducing the city’s overall sustained resilience trajectory. This observation is consistent with recent studies on the ramifications following the collapse of Baltimore’s Francis Scott Key Bridge, which demonstrated that infrastructure disruptions can generate prolonged social and economic consequences^81^. Thus, sustained resilience is not a composite index combining independent metrics, but a complementary lifecycle-based decision-support framework that provides a common quantitative basis for evaluating the coupled evolution of functionality, environmental performance, economic viability and social continuity across time.

Figure 2 describes eight (***j*** = 0–7) sustained resilience archetypes for the built environment, with archetype zero {0} being the idealised case, while the rest describe realistic life pathways. The purpose of these archetypes is to provide universal, abstract but also recognisable, generative patterns that can be reproduced for any asset, system of assets and services of the built environment, while maintaining their descriptive storytelling. Inside these archetypes, the yellow circle indicates an unacceptable level of sustained resilience—a threshold, below which, the system performance is considered inadequate. Surrounding that, we define three tiers for : Bronze, Silver and Gold tier, with the last one representing the highest or ‘golden standard’ of sustained resilience. Designers and decision makers may select Bronze, Silver, or Gold sustained resilience standards based on performance objectives, while regulatory or contextual demands may require elevated resilience levels. Unlike event-based rating systems that focus on post-shock recovery times^14^, these tiers represent cumulative lifecycle performance regimes, distinguishing minimum continuity from adaptive stability and transformative uplift across successive disruption cycles. We subsequently define the following archetypes A_k_, where k ∈{0,…,7}:

**{0} Idealised sustained resilience (****)**. This is an idealised, baseline representation of sustained resilience depicted by the solid black circle. This represents a theoretical or ‘streamlined’ benchmark scenario where the system (building, district, city) maintains its sustained resilience performance perfectly over time without any reductions or fluctuations. In this idealised scenario, we assume no deterioration or impact from hazards over the lifecycle or epoch, purely to establish a baseline. The general Eq. (1) for quantifying sustained resilience, , can be applied to various metrics that reflect sustained performance of the asset, district or city, ***i****.* Unlike conventional resilience models confined to short time windows^67^, this formulation integrates performance over the entire building lifecycle or urban epoch (***T***_***i,j***_), representing a substantial departure toward dynamic, lifecycle-based resilience modelling.1where,

***r***_***i,j***_***(t)*** represents the value of any chosen performance indicator of sustained resilience at time ***t***, e.g. structural strength, energy savings, carbon efficiency of buildings, transport network capacity, environmental performance or social inclusivity—over the lifecycle or epoch, ***T***_***i,j***_, of the asset, district or city, ***i***.

***T***_***i,j***_ represents the lifecycle for a building, or epoch for a city ***i*** for pathway ***j***.

***t*** is the time, i.e. the lifecycle of a building or the epoch of a city.

 is the sustained resilience for the idealised and hypothetical built environment (building, network, system, district, city). The area of this circle (Fig. 2, archetype {0}) represents the baseline of sustained resilience, which doesn’t deteriorate and is not affected by threats and hazards.

***r***_***i,j***_***(t)*** = ***r***_***0***_ is constant for the idealised case, meaning that the performance is unaltered.

This flexible integral approach allows us to evaluate how well a building or infrastructure performs not just in terms of short-term resilience to shocks—expressed by e.g. Minimum Operating Capability (MOC), Minimum Sustainable Capability (MSC), or Full Operating Capability (FOC)^82^—but also as a continuous dynamic measure of sustainability and adaptive capacity across evolving technical, environmental and societal conditions. However, the built environment experiences performance drops due to hazards and wear over time, and this is described by the archetype pathways {1}–{7} below.

**{1} Deterioration leading to lifespan decrease**. Archetype (Fig. 2) illustrates how deterioration leads to a decrease in lifespan of buildings and city epochs and requires more frequent city reviews, when compared to the idealised sustained resilience trajectory. The dashed purple line shows the progressive contraction of sustained resilience caused by deterioration and degradation. The shaded area that is ‘cut out’ of the circle highlights the sustained resilience , whilst the remaining area  indicates the building or city loss of performance d(t) within its reduced lifespan or epoch, *T*_1_ < *T*_0_, as per Eq. 2:

2 $${r}_{1}\left({\rm{t}}\right)={r}_{0}\left({\rm{t}}\right)\,-\,d\left({\rm{t}}\right),\,d\left({\rm{t}}\right)\,\ge \,0$$

***r₁(t)*** represents the reducing over time performance of the asset^83^ or city^84^ under deterioration. It is the time-dependent performance function that declines gradually due to e.g. declining institutional trust, soil degradation and material ageing, or other deterioration mechanisms. It captures how the actual performance deviates from the ideal baseline ***r***_***0***_ as a result of cumulative wear and damage, causing:

***d(t)*** the deterioration (reduction) of performance over time ***t***.

***T***_***1***_ is the reduced lifespan or epoch due to deterioration of the asset or system (***T***_***1***_ < ***T***_***0***_), this is the time that the performance ***r(t)*** is equal to the minimum allowable performance, ***r***_***min***_.

**{2} Threat leading to breakdown**. Archetype {2} depicts the case of a severe threat or negative change in performance—due to a threat, that could be internal or external, climatic, natural, or anthropogenic (Supplementary Information 1)—that causes a built environment breakdown, i.e. performance lower than the bare minimum, i.e. ***r***_***2***_ ***<*** ***r***_***min***_. The dashed purple trajectory illustrates the sudden loss Δ*(t)* of sustained resilience (Eq. 3), with the area of the shaded segment indicating the reduced value of the asset’s sustained resilience (). The difference from the baseline  demonstrates the lost sustained resilience compared to the baseline indicating a significant reduction. As a result, the system provides an acceptable performance level up until ***t*** = ***T***_***2***_, while it is no longer able to continue providing service, hence, its ‘as-designed’ lifespan is prematurely terminated, e.g. failure of a building due to wildfire, e.g. Los Angeles, 2025, or shelling of a city during armed conflict, e.g. Gaza, 2025.

3 $${r}_{2}\left(t\right)=\left\{\begin{array}{c}\begin{array}{cc}{r}_{0}\left(t\right), & 0\le t < {T}_{2}\\ {r}_{0}\left(t\right)-\Delta (t), & t={T}_{2}\end{array}\end{array}\right.$$

The drop ***Δ***_***2***_ determines the loss of sustained resilience due to the threat. This can be calculated by fragility models for infrastructure assets and buildings^85^ and interruption of city infrastructure services due to conflict^33,82^.

{3} Proactive increase of sustained resilience leading to lifecycle prolongation

**Fig. 3:**
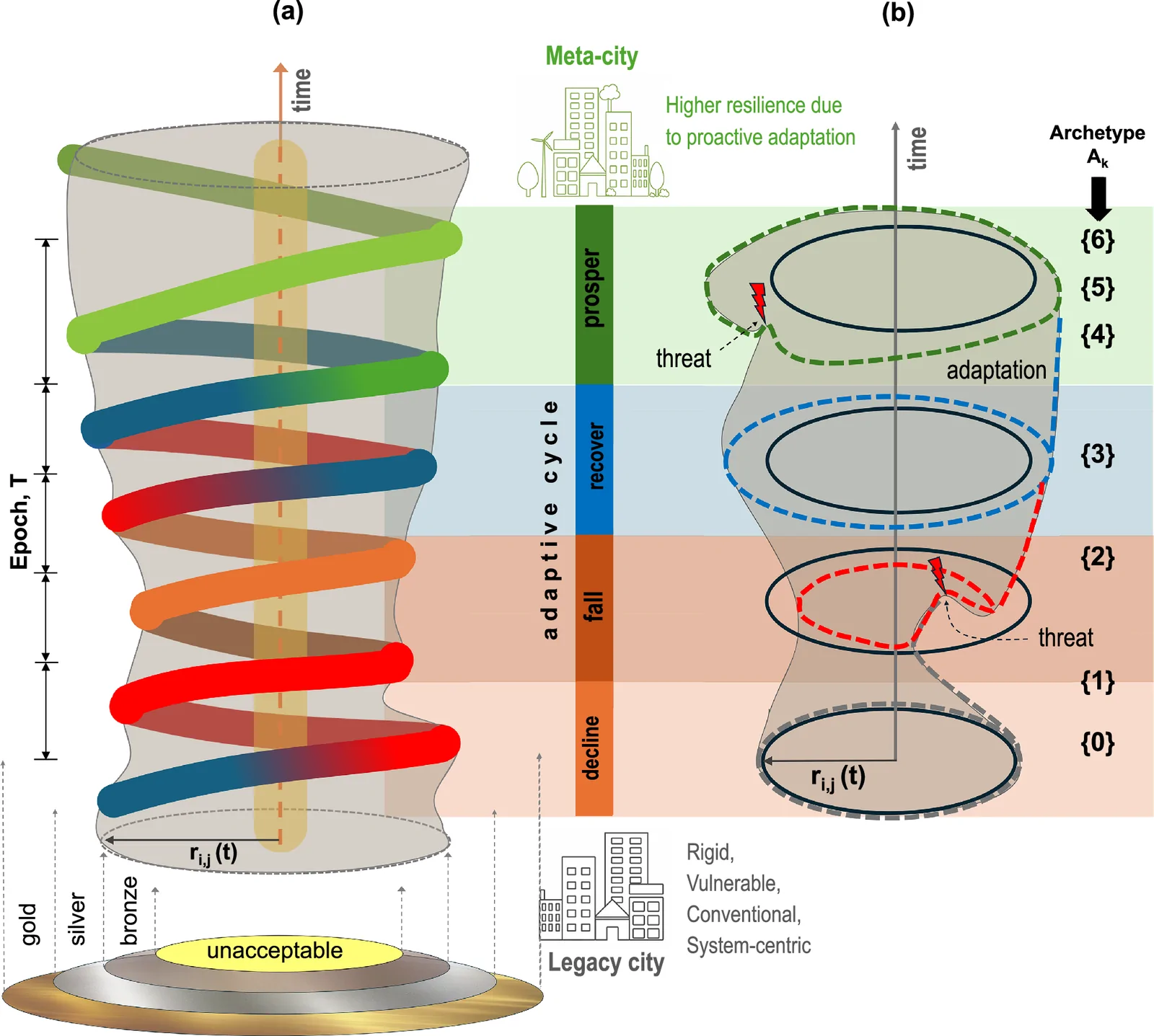
Sustained resilience of cities across epochs and archetypal pathways. **a** Sustained resilience  conceptualised as a time-integrated, cross-epoch property of cities. Each epoch captures how cities evolve under internal dynamics and external pressures. These epochs are governed by formal policy and planning cycles (Box 1), spanning long-range strategic frameworks (≈10–15 years), statutory plan revisions (≈3–5 years), and annual or quarterly performance reviews that define the cadence of regulatory adjustment and investment allocation. The grey volume represents the measure of sustained resilience: larger volumes indicate greater cumulative capacity to withstand, recover, adapt and evolve over the urban lifecycle. The helical trajectory illustrates the temporal integration of performance, r_i,j_(t), across epochs (T_i,j_), transitioning through decline, fall, recovery and prosper phases. The legacy city at the base follows a rigid, system-centric trajectory with limited adaptive gain, typically confined to lower sustained resilience tiers (e.g. Bronze), whereas the meta-city at the top represents an idealised future condition in which sustained resilience has been systematically enhanced across successive epochs, aligning long-term urban performance with urban strategies. **b** Archetypal pathways of sustained resilience across urban epochs. Dashed trajectories represent departures from baseline performance due to threats, recovery strategies, adaptation, or proactive upgrading. Divergent pathways emerge as cities experience shocks, policy shifts, or proactive interventions. Upward-widening trajectories reflect proactive adaptation and learning, enabling progression toward higher sustained resilience tiers (e.g. Silver and Gold), while narrowing trajectories signify stagnation, regulatory obsolescence, or decline. The decline–fall–recover–prosper sequence is conceptually compatible with adaptive-cycle theory, but sustained resilience translates this logic into cumulative lifecycle performance across successive urban epochs. Archetypes {0}–{6} correspond to the pathways introduced in Fig. 2, illustrating how cities may transition between sustained resilience states through time. The coloured background bands denote successive phases of the adaptive cycle (decline, fall, recover, prosper). Solid black ellipses represent baseline sustained resilience states, while dashed coloured trajectories indicate evolving archetypal pathways. The symbol r_i,j_(t) denotes time-dependent performance, T denotes the duration of an epoch and the vertical axis represents time.

In this archetype, sustained resilience is proactively uplifted by ***u(t)*** at time ***t***_***3***_ (see Eq. 4) to reach the golden standard (Tier 3). This strategic investment prolongs the lifespan of the built environment beyond its original design one (***T***_***3***_ > ***T***_***0***_). This is represented by the expanded dashed triangle and additional shaded area extending outside the baseline circle, showing the gains in sustained resilience and lifecycle. In cities, this is shown with an expansion of the volume of the helix (‘prosper’ in Fig. 3) representing a period of growth and improved quality of services.

4 $${r}_{3}\left(t\right)\,=\,\left\{\begin{array}{cc}{r}_{0}\left(t\right), & 0\,\le \,t\, < \,{t}_{3}\\ {r}_{0}({\rm{t}})\,+\,{\rm{u}}({\rm{t}}), & {t}_{3}\,\le \,t\,\le \,{T}_{3}\end{array}\right.$$

The duration required to achieve an uplift in sustained resilience varies substantially across scales of the built environment. While the renovation of a single building may take days to months, upgrading a city service (e.g. transport or energy networks) or an entire district through spatial interventions can extend over years or even decades. Such upgrades may involve renovating and enhancing existing components or urban areas, introducing new ones, or applying a hybrid strategy that combines both—preserving valuable existing assets and/or integrating new systems. At the city scale, the uplift function ***u(t)*** encompasses not only physical interventions or additions (e.g. new transport modes or infrastructure) but also improvements in system interdependencies and interconnectivity efficiency. Examples include establishing synergistic multimodal transport networks, enhancing redundancy and backup power systems, or optimising cross-sector coordination to reinforce urban functionality and adaptive capacity^86^.

**{4} Reactive enhancement of sustained resilience**. Here, sustained resilience is improved reactively following a disruption or shock (e.g. flash floods or political regime change influencing urban and social fabric) at ***t***_***4.1***_ that leads to a reduced performance, ***r***_***4.1***_ < ***r***_***0***_, thus the building or city suffers a drop, Δ***(t)****=*Δ_***4***_, in sustained resilience, but targeted renovations and recovery^87^ enhance performance, indicated as ***e(t)***, to a level higher than the original one, ***r***_***0***_ < ***r***_***4.2***_. The outcome may be a return to the original baseline (***r***_***0***_), an upgrade to Tier 2 (Silver—***r***_***4***.***2***_), which is what is illustrated in this archetype, or, depending on interventions, even a leap toward Tier 3 (Gold). The shaded area illustrates the sustained resilience , which is the result of r_4_ fluctuating throughout the lifecycle of the built environment. It is generally assumed that the target performance level following recovery or enhancement will not fall below the original baseline condition, ***r***_***0***_.

5 $${r}_{4}(t)\,=\left\{\begin{array}{ll}{r}_{0}(t),\\ {r}_{4.1}\,=\,{r}_{0}({\rm{t}})\,-\,{\rm\Delta }(t),\\ {{r}_{4.2}=r}_{0}({\rm{t}})\,-\,{\rm\Delta }(t)\,+\,e(t),\\ {r}_{4.2}({\rm{t}}),\end{array}\right.\begin{array}{ll}0\,\le \,t\, < \,{t}_{4.1}\\ {t=t}_{4.1}\\ {t}_{4.1}\, < \,t\,\le \,{t}_{4.2}\\ {t}_{4.2}\, < \,t\,\le \,{T}_{4}\end{array}$$

**{5} Restoration and adaptation leading to lifespan or epoch prolongation**. Here, the performance is prolonged to ***T***_***5***_ through iterative cycles of performance loss (***Δ***_***5***_) at time ***t***_***5.1***_, and performance elevation, ***e(t)****,* from ***r***_***5.2***_ onwards. This can be modelled with the same rationale as per archetype {4} above. Similarly, the uplift ***u(t)*** from ***r***_***5.3***_ and lifespan or epoch extension to ***T***_***5***_ can be modelled as per archetype {3}. This is a combination of loss and gain of  throughout the lifecycle. For example, a port city repeatedly affected by flooding may follow this archetype when each flood is followed not only by repair, but also by incremental adaptation, such as raised utilities, nature-based solutions for drainage, and wetland restoration and upgraded evacuation routes, progressively extending the district’s functional urban epoch.

**{6} Regulatory uplift and {7} Demand or importance reduction**. In these archetypes, the target tier of sustained resilience remains constant (Bronze) over its lifecycle, similar to the idealised model shown in the first figure. The key difference here is that regulatory requirements and design standards, politics or global strategies have been uplifted over time {6} (e.g. the amendment of design guidelines) or demand and importance have declined {7} (e.g. due to population displacement during a war), demonstrating the dynamic performance demand throughout the lifecycle. In archetype {6} the built environment was originally compliant and positioned, for example, between the Bronze and Silver tiers at design, now falls into the obsolete or unacceptable range—the yellow area—because the standards and goals it was built to meet are no longer sufficient. In other words, the built environment itself is not underperforming relative to its original design. Rather, it has become outdated due to new, more stringent requirements. This necessitates renovation of assets and/or upgrade of the city services up to the new standards. At a certain point in time, ***t***_***6***_, the required performance standard increases. As a result, the overall sustained resilience—represented by —is effectively increased . This regulatory uplift—whether driven by new regulations, heightened end-user expectations, or increased stakeholder and owner requirements—can also be modelled as per archetype {3}. The difference here is that we have not modelled the extension of the lifespan while thresholds of the sustained resilience tiers have increased. The opposite is valid for archetype {7} where decline in demand, e.g. reduction of traffic over a bridge, urban depopulation or repurposing, causes a reduction in importance, meaning that tier thresholds contract, and accordingly  < .

The archetypes of sustained resilience provide a foundational seamless integration and operationalisation of a tiered lifecycle model that normalises resilience and sustainability in the temporal domain. The archetypes are not intended as deterministic or mutually exclusive pathways; rather, they provide a generative basis for representing realistic scenarios by superimposing or sequencing into confluent archetypes across temporal scales. For example, Archetypes {1} + {4} may describe an ageing city whose infrastructure and institutional capacity have gradually deteriorated before an acute flood causes sudden performance loss, followed by reactive recovery. Another example, where Archetypes {1} + {3} + {6} interact, may describe an old neighbourhood that first experiences long-term decline, is then proactively upgraded and renewed, and subsequently becomes subject to a higher minimum performance threshold introduced through new policy or regulation.

In summary, the shaded areas across all archetypes represent varying measures of sustained resilience over the lifecycle of a structure. In some scenarios, temporal sustained resilience is lower than the idealised baseline shown in the baseline archetype, indicating performance losses or obsolescence. In other cases—such as with proactive advancements or restoration and adaptation pathways—the lifecycle of the built environment is enhanced. For all archetypes, the threshold of minimum acceptable sustained resilience is not defined by a momentary performance condition over a short recovery window, as is often the case in conventional resilience assessments, i.e. *r*(t) > *r*_b_ = *r*_min_. Rather, it reflects performance sustained across extended temporal horizons during which buildings, infrastructure, and cities are expected to maintain functionality, adapt and apply sustainable practices. These horizons may range from months and years to full asset lifecycles and urban epochs. Consequently, sustained resilience  should remain greater than or equal to a predetermined lifecycle-performance threshold, corresponding to the selected tier of performance . Relative to the baseline sustained resilience, , the sustained resilience can be classified as superior when it increases over time , stagnant when it remains at the baseline level  or inferior when regression occurs during the lifecycle .

### Upscaling sustained resilience to systems and cities

Figure 3 conceptualises sustained resilience as a time-integrated, cross-epoch property of cities. Moving upward in time, cities transition through phases of decline, fall, recovery, and prosper, following distinct archetypal pathways across sustained resilience tiers (Bronze to Gold). The expanding or contracting volume represents cumulative sustained resilience, distinguishing rigid legacy cities from adaptive meta-cities shaped by proactive, policy-driven adaptation.

A top-down approach to sustained resilience can be anchored in planetary boundaries^11,12^, where global sustainability thresholds—such as limiting global temperature rise to 2 °C—are interpreted not only as environmental limits but also in terms of socially acceptable resilience outcomes. For example, such a threshold could be associated with explicit resilience constraints, such as affecting fewer than ten major cities or fewer than 100 million people worldwide. These global constraints can then serve as sustained-resilience reference boundaries that are systematically decomposed into performance requirements at city and district scales. Conversely, sustained-resilience criteria defined at the city scale can be disaggregated bottom-up into measurable requirements for buildings, infrastructure networks, services and operational systems, enabling vertical alignment of resilience objectives across scales. In this way, sustained resilience extends beyond conventional planetary boundaries focused on environmental limits alone, toward synthetic sustained-resilience boundaries that explicitly recognise the coupled protection of the planet, people and biodiversity and the built environment.

### Operationalisation and implementation challenges of sustained resilience

Sustained resilience is intended as a decision-support framework for planning, asset management and renewal, and urban governance. The framework builds upon familiar metrics across social, ecological, technological, economic and governance domains, including functionality, accessibility, service continuity, emissions, lifecycle costs, ecosystem services and social inclusion. These metrics are redefined to evaluate cumulative performance across disturbance, recovery, adaptation and renewal cycles over extended lifecycles. Consequently, sustained resilience assesses long-term trajectories of these metrics, rather than their condition at a single point in time. The evaluation is performed over successive planning epochs (Box 1) and integrated through the proposed mathematical formulation to derive sustained resilience trajectories for assets, services, infrastructure systems and cities. The archetypes provide a diagnostic tool for identifying whether systems are following deteriorating, recovering, adaptive, prospering, or declining periods and pathways. The Bronze, Silver and Gold tiers provide transparent performance targets and minimum acceptable thresholds which are set by the city stakeholders.

Thus, the framework is operationalised for decisions to enable the comparison of alternative interventions, which are likely to result in deteriorating, recovering, adaptive, or prospering sustained resilience trajectories, thereby supporting transparent investment prioritisation and planning. The framework does not prescribe universal weights or thresholds. Further guidance on weighting, prioritisation and the aggregation of sustained resilience across domains and scales is provided in Box 1. Similarly, uncertainty, competing objectives and value conflicts are recognised as inherent features of urban decision-making. These may be addressed through, for example, scenario and sensitivity analyses, probabilistic modelling, multi-criteria decision-making methods^88^ and adaptive governance approaches. The application of the framework also presents challenges. Cross-scale aggregation inevitably introduces conceptual, methodological and governance complexities. Indicators may differ in their spatial and temporal resolution, data availability, quality, uncertainty and institutional ownership. Long-term assessment of sustained resilience trajectories is also subject to uncertainty associated with future hazards, climate change, socio-economic developments, technological evolution and governance conditions. In addition, some dimensions of sustained resilience, such as social cohesion, institutional trust, cultural values, wellbeing and equity, remain inherently difficult to quantify. Thus, they require harmonisation through non-dimensionalisation, probabilistic assessment and multi-criteria aggregation methods. The governance authority, stakeholder priorities and system boundaries often vary across assets, communities and cities. Consequently, the proposed aggregation should not be interpreted as reducing urban complexity to a single objective function. Rather, sustained resilience provides a common assessment space within which heterogeneous social, ecological, technological, economic and governance trajectories can be evaluated, aggregated and disaggregated across scales. Convergence of top-down policy objectives and bottom-up participatory processes remains essential for managing competing priorities, contested values, and uneven social outcomes, while methods such as scenario analysis, reverse stress testing^89^, sensitivity analysis and adaptive governance can support more robust and equitable decision-making. Thus, research will be needed to shed light on uncertainty quantification, participatory weighting, adaptive governance and cross-scale assessment methods for sustained resilience.

Box 1. Upscaling sustained resilience from domains to cities, continents and the planet Sustained resilience  is inherently scalable and systemic. It is an aggregated decision-support property that integrates performance across time (lifecycle/epochs), space (assets → cities → countries → planet) and domains. At the city scale, the sustained resilience of each urban domain—built and natural environment, infrastructure and services, people and communities, economy and governance and their interdependencies—is quantified as a domain-level property (, , …). These domains align with a SETS perspective, whereby people and communities represent the social system, the built and natural environment and infrastructure represent ecological and technological systems, and economy and governance provide the institutional mechanisms coordinating interactions across scales. Each domain aggregates multiple sub-components (e.g.  , , ) synthesised through weighted aggregation to reflect local priorities, policy objectives and risk profiles.

**Figure:**
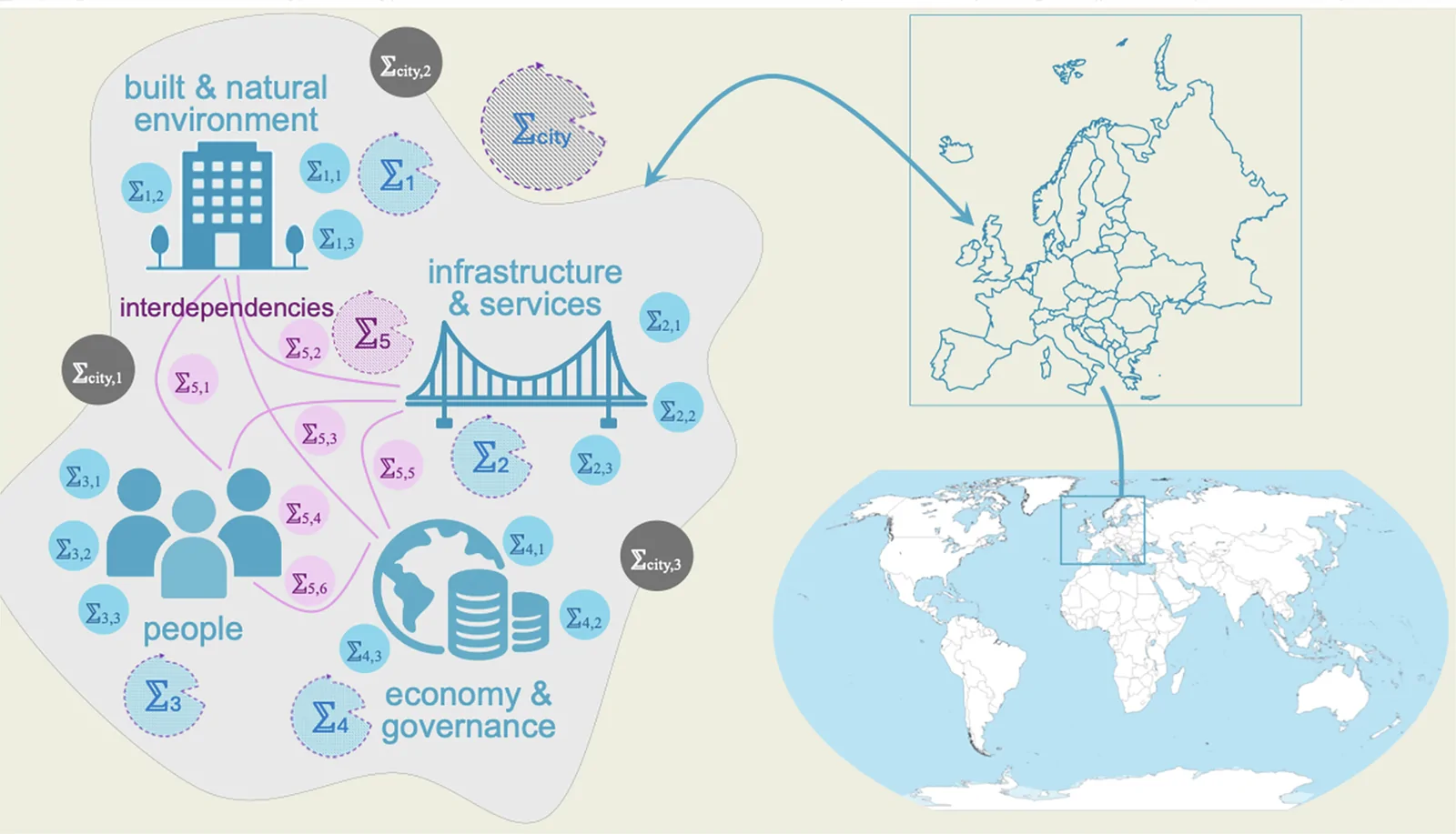

Crucially, weights are not fixed. Cities may prioritise different sustained resilience tiers (Bronze, Silver, Gold) across different time horizons, and hence, experience different archetypal pathways depending on strategic intent and threat occurrence. Thresholds and weights may be determined through local policy objectives, stakeholder participation, regulatory requirements and risk-informed decision processes. Advanced multi-criteria optimisation frameworks, including AI dashboards integrating fuzzy logic, TOPSIS, evolutionary algorithms and reinforcement learning, enable cities to dynamically re-weight objectives—such as emissions, travel time and social access—and steer domain performance toward targeted sustained resilience trajectories. By convolving sustained resilience across all domains ( to ) and their interdependencies , the framework quantifies the total sustained resilience of a city  for defined spatial boundaries and planning epochs. Tracking  across successive epochs enables cities to diagnose whether they are following archetypes of e.g. proactive advancement or decline, and to intervene through targeted investments, policy reform, or system upgrades. Importantly, city-level optimisation can propagate downward, reshaping sustained resilience requirements at domain and asset levels. The same logic extends beyond the city. Sustained resilience can be aggregated across cities to national, continental and global scales, aligning with planetary boundaries and the Sustainable Development Goals.

**Table:** 

Scale	Description	Decision levers examples	Conceptual equation for
**Component**	Sustained resilience performance (r_i,j_) of individual components (e.g. buildings, transport links, services), quantified over lifecycle or epoch (T_i,j_)	Design guidelines, materials, operation, maintenance, use and repurposing	Eq. (1)
**Domain/district**	Aggregated sustained resilience of a city domain/district (e.g. built environment, infrastructure, communities), combining multiple components	Policy priorities, regulatory sustained resilience thresholds	$\sum {w}_{i,j}=1$, n_i_: number of r_i,j_ (6)
**City**	Total sustained resilience of a city/building stock within defined spatial boundaries and planning epochs (T_i,j_)	Urban strategy, investment timing, optimisation objectives, renewal	d: number of city domains and interdependencies (7)
**National/Continental**	Aggregated sustained resilience across multiple cities or systems within geopolitical boundaries	National policy, climate commitments, resource allocation	N: number of cities (8)
**Global**	Integrated sustained resilience subject to planetary boundaries	Global governance, SDGs, equity constraints, climate targets	R: number of regions (9)
where, r_i,j_(t) represents time-varying performance (e.g. service, emissions efficiency, accessibility). w_i,j_(t), α_k_ and β_r_ are dynamic, policy- and risk-dependent weights, enabling optimisation and re-prioritisation across tiers and archetypes. ${\mathcal{C}}$(^.^) denotes convolution, capturing interdependencies, trade-offs and cascading effects across domains and time.

## Conclusions

We contend that continuing to treat resilience and sustainability as parallel, loosely connected agendas is no longer tenable in an era of escalating climate volatility, infrastructure ageing and compounding, threat-agnostic disruptions. Treating resilience as short-term recovery and sustainability as long-term efficiency creates structural policy blind spots, incompatible metrics across spatial and temporal scales and fragmented governance across services and sectors. We therefore introduce sustained resilience as a new, unifying property of cities and the built environment—one that measures performance seamlessly across time, space, services and tiers, from buildings (micro) to districts (meso), cities and stocks (macro) and across full lifecycles and urban epochs. Sustained resilience extends existing resilience and sustainability research by providing a lifecycle-oriented quantitative framing that complements established concepts such as Panarchy, SETS and adaptive resilience. Here, resilience and sustainability are reframed as inseparable, lifecycle-integrated dimensions, formalised into a single, cumulative and adaptive performance property. This interpretation is complementary to Panarchy and recent people-centric urban resilience frameworks that conceptualise cities through the interdependent domains of society, economy and the natural and built environment. Sustained resilience is intended to support, rather than replace, context-specific governance and deliberation. Although the framework provides a quantitative, lifecycle-oriented basis for assessing resilience, quantitative metrics are designed to inform decisions rather than automate them. Social, cultural, institutional and justice-related dimensions cannot be fully reduced to a single performance indicator without risking the loss of context, contested values and minority perspectives. Consequently, the selection, weighting and interpretation of indicators should be undertaken through transparent stakeholder engagement and context-sensitive governance, ensuring that resilience investments remain aligned with societal priorities rather than performance metrics alone.

The framing can be applied across systems and services and encompasses archetypal pathways of sustained resilience, creating a common language for engineers, planners, policymakers and communities. Operationally, it provides a lifecycle-aware, policy-actionable methodology that integrates short-term shock response, medium-term recovery and adaptation, and long-term transformation. The archetypes described here illustrate pathways of performance and a measurable mathematical structure for converging bottom-up and top-down policy targets and decision making to guide investment. The proposed archetypes transform sustained resilience from a fixed classification into a generative modelling language, enabling realistic urban trajectories to be represented by sequencing or superimposing slow deterioration, acute disruption, proactive renewal, and regulatory uplift across multiple temporal scales across performance tiers, whose thresholds evolve with regulatory uplift, demand shifts, and societal expectations. In doing so, sustained resilience moves beyond the aggregation of separate metrics to provide a lifecycle-oriented decision-support framework for evaluating alternative development pathways, investments and adaptation strategies across changing temporal, spatial and service regimes, enabling cities not only to withstand shocks but also to adapt, evolve and prosper over time.

## Supplementary information

Supplementary Information

## Data Availability

No datasets were generated or analysed during the current study.
