# Supplementary material for: Sustained resilience as a unifying property of cities and the built environment
Source: NPJ Urban Sustain. 2026 Aug 25;6(1):114. doi: 10.1038/s42949-026-00457-3 (PMC13518238; doi:10.1038/s42949-026-00457-3)
Supplement: Supplementary file 1 [file 42949_2026_457_MOESM1_ESM.docx]

npj | urban sustainability	**Perspective**

Supplementary Information 1

**Sustained resilience as a unifying property of cities and the built environment**

**Stergios-Aristoteles Mitoulis**^1,3,4 *^**, Nadiia Kopiika**^1,3,4^**, Roberta Di Bari**^1,3^**, Sotirios Argyroudis** ^2,3,4^

^1^ Centre for Global Infrastructure Resilience, The Bartlett School of Sustainable Construction, UCL, London, United Kingdom

^2^ Brunel University of London, Uxbridge, United Kingdom

^3^ [www.MetaInfrastructure.org](https://www.MetaInfrastructure.org), London, United Kingdom

^4^ [www.BridgeUkraine.org](https://www.BridgeUkraine.org), London, United Kingdom

*corresponding author: S-A Mitoulis, [S.Mitoulis@UCL.ac.uk](mailto:S.Mitoulis@UCL.ac.uk)

Terminology for disturbances and forces of change in sustained resilience

The sustained-resilience framework covers acute events, slowly accumulating pressures, and changes in the conditions or performance requirements of built-environment systems (Fig. S1). To provide a consistent terminology, the manuscript distinguishes (i) potential sources of harm, (ii) realised disturbances and system-level consequences, and (iii) longer-term contextual changes that modify baseline conditions and performance expectations. Figure S1 illustrates the relationships among these concepts, while Table S1 provides the operational definitions adopted throughout the manuscript. These definitions are informed by established disaster-risk, critical-infrastructure, and resilience terminology [1,2]

Supplementary Table S1 | Operational terminology used in the sustained-resilience framework

| **Term** | **Operational meaning in this manuscript** | **Performance/time signature** | **Illustrative examples and recommended use** |
| --- | --- | --- | --- |
| **Hazard** | A process, phenomenon or human activity with the potential to cause harm. A hazard is a potential source of adverse effects, not the performance loss itself [1,2]. | Potential or recurring; may be acute or slow-onset. | Earthquake, flood, wildfire, extreme heat, technological failure mechanism. Use “multi-hazard” only when several potential damaging phenomena are considered. |
| **Threat** | A potential source or circumstance of harm, reserved here mainly for deliberate, adversarial, security-related or explicitly threat-framed contexts. It should not be the default umbrella for all forms of change [7, 9, 10]. | Potential; may be acute, sustained or strategic. | Armed attack, cyberattack, terrorism or hostile interference. Conflict may act as a threat, a prolonged stress or a wider crisis, depending on scale and duration. |
| **Shock** | A realised acute disturbance that produces an abrupt deviation or loss in system performance. A shock may result from a hazard or threat but is conceptually distinct from the source [7-9] | Sudden onset; seconds to weeks; discontinuity in r(t). | Earthquake impact, flash flood, cyber outage, sudden bridge closure, abrupt policy or market interruption. |
| **Chronic stress** | A persistent, recurrent or cumulative pressure that gradually erodes functionality, capacity, equity or adaptive resources [7,8]. | Slow or repeated onset; months to decades; progressive change in r(t). | Material deterioration, ageing, congestion, heat exposure, inequality, institutional erosion, recurrent drought or maintenance backlog. |
| **Crisis** | A serious system-level condition of disruption or instability requiring strategic coordination. It may emerge from one severe shock or from interacting shocks and chronic stresses and may propagate through interdependencies [3-6]. | Weeks to years; nonlinear escalation, cascading effects or prolonged impairment. | Energy, humanitarian, public-health or infrastructure crisis; cascading disruption across transport, power, water and communities. |
| **Long-term force of change** | A gradual structural driver that modifies baseline conditions, exposure, vulnerability, demand, priorities or acceptable performance thresholds, without necessarily causing immediate damage [1,2,8]. | Years to decades; shifts baselines, trajectories or tier thresholds. | Climate change, demographic transition, urbanisation, technological transition, institutional change and geopolitical restructuring. Climate change is treated primarily as a driver that modifies hazards and stresses. |
| **Regulatory or demand shift** | A specific force of change that alters the required or valued level of performance rather than physically damaging the system [7,9] | Discrete or gradual threshold change; modifies rmin or tier boundaries. | Stricter design codes, decarbonisation targets, changing service demand, depopulation, repurposing or increased societal expectations. |
| **Disruption** | A neutral outcome term denoting an interruption, degradation or deviation in system performance caused by one or more shocks, stresses, crises or forces of change [1,7]. | Observed effect in r(t), rather than a causal category. | Service interruption, reduced accessibility, loss of capacity or institutional dysfunction. Replace broad phrases such as “threat-agnostic disruptions” with the specific categories being considered. |

**Interpretive note.** The categories are related but not mutually exclusive. For example, climate change is a long-term force of change that modifies the frequency or intensity of hazards and chronic stresses; conflict may be framed as a deliberate threat, a prolonged stress or a system-wide crisis; and deterioration is an endogenous chronic stress rather than an external hazard. The framework quantifies their realised consequences through changes in time-dependent performance, r(t).

**Figure S1 | Relationships among drivers, disturbances and realised sustained resilience.** Long-term forces of change (e.g. climate, demographic, technological, economic, governance, land-use, and social change) shape baseline system conditions and exposure, influencing the occurrence and evolution of hazards, threats, shocks and chronic stresses. These disturbance types interact and may escalate into system-wide crises, while regulatory and demand shifts modify required performance thresholds through changes in policy, standards and societal expectations rather than directly causing physical damage. The overlapping regions highlight that these categories are not mutually exclusive and may coexist, evolve or transition depending on context, spatial scale and duration. The lower panel conceptualises **realised sustained resilience** as the temporal evolution of system performance, r(t), capturing the cumulative effects of interacting disturbances, changing performance requirements and adaptive responses over time.

**References**

1. United Nations Office for Disaster Risk Reduction (UNDRR). Sendai Framework Terminology on Disaster Risk Reduction (UNDRR, Geneva, 2017; continuously maintained online).

2. United Nations Office for Disaster Risk Reduction & International Science Council. Hazard Information Profiles (HIPs): 2025 version (UNDRR and ISC, Geneva and Paris, 2025).

3. Alexander, D. E. Resilience and disaster risk reduction: an etymological journey. Nat. Hazards Earth Syst. Sci. 13, 2707–2716 (2013). <https://doi.org/10.5194/nhess-13-2707-2013>

4. Pescaroli, G. & Alexander, D. A definition of cascading disasters and cascading effects: going beyond the “toppling dominos” metaphor. Planet@Risk 3, 58–67 (2015).

5. Pescaroli, G. & Alexander, D. Critical infrastructure, panarchies and the vulnerability paths of cascading disasters. Nat. Hazards 82, 175–192 (2016). <https://doi.org/10.1007/s11069-016-2186-3>

6. Alexander, D. E. A magnitude scale for cascading disasters. Int. J. Disaster Risk Reduct. 30, 180–185 (2018). https://doi.org/10.1016/j.ijdrr.2018.03.006

7. Mentges, A., Halekotte, L., Schneider, M., Demmer, T. & Lichte, D. A resilience glossary shaped by context: reviewing resilience-related terms for critical infrastructures. Int. J. Disaster Risk Reduct. 96, 103893 (2023). <https://doi.org/10.1016/j.ijdrr.2023.103893>

8. Belloni, G. et al. Health systems governance, shocks and resilience: a scoping review of key concepts and theories. BMJ Glob. Health 10, e017358 (2025). <https://doi.org/10.1136/bmjgh-2024-017358>

9. International Organization for Standardization. ISO 22300:2025, Security and resilience — Vocabulary (ISO, Geneva, 2025).

10. Trump, B. D. et al. Threat-agnostic resilience: framing and applications. Int. J. Disaster Risk Reduct. 124, 105535 (2025). <https://doi.org/10.1016/j.ijdrr.2025.105535>
